# Counter‐Intuitive Gas‐Phase Reactivities of [V_2_]^+^ and [V_2_O]^+^ towards CO_2_ Reduction: Insight from Electronic Structure Calculations

**DOI:** 10.1002/anie.202001223

**Published:** 2020-03-25

**Authors:** Jilai Li, Caiyun Geng, Thomas Weiske, Helmut Schwarz

**Affiliations:** ^1^ Institut für Chemie Technische Universität Berlin 10623 Berlin Germany; ^2^ Institute of Theoretical Chemistry Jilin University 130023 Changchun China

**Keywords:** CO_2_ activation, gas-phase reactions, oxidation state, quantum chemical calculations

## Abstract

[V_2_O]^+^ remains “invisible” in the thermal gas‐phase reaction of bare [V_2_]^+^ with CO_2_ giving rise to [V_2_O_2_]^+^; this is because the [V_2_O]^+^ intermediate is being consumed more than 230 times faster than it is generated. However, the fleeting existence of [V_2_O]^+^ and its involvement in the [V_2_]^+^ → [V_2_O_2_]^+^ chemistry are demonstrated by a cross‐over labeling experiment with a 1:1 mixture of C^16^O_2_/C^18^O_2_, generating the product ions [V_2_^16^O_2_]^+^, [V_2_^16^O^18^O]^+^, and [V_2_^18^O_2_]^+^ in a 1:2:1 ratio. Density functional theory (DFT) calculations help to understand the remarkable and unexpected reactivity differences of [V_2_]^+^ versus [V_2_O]^+^ towards CO_2_.

## Graphical abstract

**The fleeting existence** of [V_2_O]^+^ as a reactive intermediate in the oxidation of [V_2_]^+^ by CO_2_ to form [V_2_O_2_]^+^ has been identified; the counter‐intuitive oxidation behavior of [V_2_]^+^ versus [V_2_O]^+^ and the quite enormous reactivity differences have been traced back to the supportive role of the bridging oxygen atom in [V_2_O]^+^.

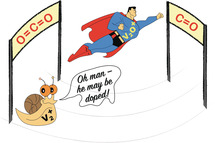

Rolf Huisgen has shaped physical organic chemistry in a nearly unparalleled fashion.^1^ His extensive, seminal work on, for example, 1,3‐dipolar cycloadditions1b forms a pillar of contemporary mechanistic organic chemistry, not to speak of their role in the synthesis of heterocyclic compounds. By using the then available experimental repertoire, the Huisgen school unraveled the finest mechanistic details of numerous chemical processes, and their work gives testament for chemist generations to come.^2^

A familiar kinetic scheme of a multi‐step process is depicted in Equation 1, and one problem here concerns the identification of “intermediate” [B]. This holds true in particular when the rate constants *k*_1_ and *k*_2_ differ significantly, that is, *k*_2_ ≫ *k*_1_.
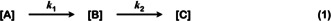



A typical example is provided by the gas‐phase chemistry of the [Pd]^+^/CH_3_I couple which gives rise to the formation of [Pd(CH_2_I)]^+^.^3^ In this case, the product ion is not formed by the entropically favored direct cleavage of the C−H bond of CH_3_I.3a Rather, in the first step a short lived [Pd(CH_3_)]^+^ intermediate is generated, which reacts further with CH_3_I in a very fast metathesis process to form [Pd(CH_2_I)]^+^ and CH_4_.3b Recently, in the context of combined experimental/computational studies on the timely topic of CO_2_ activation,^4^ we came across a similar, unexpected situation in the thermal gas‐phase reactions of bare diatomic [V_2_]^+^ with CO_2_.

The cluster [V_2_]^+^ was formed by supersonic expansion of helium into a vanadium plasma generated by laser ablation/ionization of a vanadium disk using a Nd:YAG laser, operating at 532 nm inside the external cluster source of a Fourier‐transform ion cyclotron resonance (FT‐ICR) mass spectrometer as described previously (for details, see the Supporting Information).^5^ A fraction of the ion population was then guided by a static ion optical system into the ICR cell. Next, in a sequence of pulses, argon was admitted to the ICR cell such that the ions collide on average about 1×10^5^ times with argon. This procedure ensures thermalization of hot ions and quenching of excited electronic states. After mass‐selection of the thermalized [V_2_]^+^ ions, they were reacted with CO_2_ or C^18^O_2_ at a constant pressure low enough to ensure single collision conditions. The elementary compositions of the charged species have been confirmed by exact mass measurements using high resolution mass spectrometry.

The result of the reaction of [V_2_]^+^ with CO_2_ at a partial pressure of 1.0×10^−7^ mbar and a reaction time of 10 s inside the ion trap of the FT‐ICR machine is shown in Figure 1 b. In addition to the signal of the starting material [V_2_]^+^ (signal **A**, Figure 1), a signal **D** appears, which has been assigned the formula [V_2_O_2_]^+^ by exact mass measurements; also, very small amounts of atomic [V]^+^ are present (signal **B**). Formally, the formation of [V_2_O_2_]^+^ corresponds to the loss of one carbon atom. If C^18^O_2_ acts as a reaction partner, signal **D** from Figure 1 b is shifted by four mass units (Figure 1 c; signal **E**). Clearly, oxygen atom transfer (OAT) from CO_2_ to the vanadium cluster takes place. However, since a carbon atom represents an extremely poor leaving group, the suggestion was raised as to whether the formation of [V_2_O_2_]^+^ from the [V_2_]^+^/CO_2_ couple is the result of a multi‐step process, although the spectra in Figure 1 b,c do not exhibit any clue for the involvement of conceivable intermediates. The assumption for the operation of a multi‐step process is corroborated by a crossover‐labeling experiment using a 1:1 mixture of C^16^O_2_ and C^18^O_2_: The appearance of [V_2_^16^O^18^O]^+^ (signal **F** in Figure 1 d) together with the other two isotopomers in a 1:2:1 ratio verifies this hypothesis. According to Equations (2) and (3) and in line with Figure 1 d, one has to conclude that [V_2_]^+^ reacts consecutively with two CO_2_ molecules via an OAT by the release of one CO molecule each time; the monoxide [V_2_O]^+^ serves as a short‐lived intermediate (Scheme 1). Further oxidation processes according to Equation (4) in Scheme 1 do not occur at ambient temperature.

**Figure 1:**
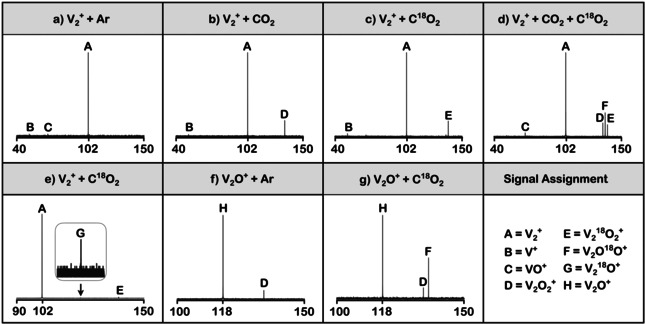
Representative mass spectra: a)–e) are for the thermal reactions of [V_2_]^+^ with a) Ar at 1.0×10^−7^ mbar, b) C^16^O_2_ at 1.0×10^−7^ mbar, c) C^18^O_2_ at 1.0×10^−7^ mbar, and d) a 1:1 mixture of C^16^O_2_ and C^18^O_2_ at 2.0×10^−7^ mbar after a reaction time of 10 s, respectively; e) C^18^O_2_ at 1.0×10^−7^ mbar and a reaction time of 1s; f) and g) represent the thermal reactions of [V_2_O]^+^ with f) Ar at 4.0×10^−9^ mbar, g) C^18^O_2_ at 4.0×10^−9^ mbar, after a reaction time of 2s, respectively. All *x*‐axes are scaled in *m*/*z*, and the *y*‐axes are normalized relative ion abundances.

**Scheme 1:**
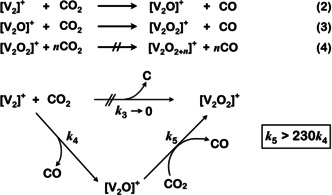
Multi‐step activation of CO_2_ by [V_2_]^+^ with [V_2_O]^+^ serving as a fleeting intermediate.

In the [V_2_]^+^/CO_2_ couple, the supposed intermediate [V_2_O]^+^ can only be detected experimentally as an extremely weak signal under highly optimized conditions of both pressure and reaction time (signal **G** in Figure 1 e). However and fortunately, [V_2_O]^+^ can be synthesized independently and in sufficient quantities in the external ion source by adding traces of O_2_ to the helium buffer gas (Figure 1 f). As shown in Figure 1 g, the so‐formed [V_2_^16^O]^+^ indeed reacts with C^18^O_2_ to produce [V_2_^16^O^18^O]^+^ by the expulsion of C^18^O. The collision efficiency^6^ *φ* of that reaction is close to 100 %. The high reactivity of [V_2_O]^+^ explains why, [V_2_O]^+^ from the reaction of [V_2_]^+^ with CO_2_, remains elusive. As soon as the monoxide has formed within the ICR cell, it immediately reacts with another CO_2_ molecule. The high reactivity of the [V_2_O]^+^ intermediate, generated from the [V_2_]^+^/CO_2_ couple, is further enhanced due to the substantial exothermicity of the process (see below) and the fact that, owing to the absence of a heat bath, part if not most of the excess energy is stored in the ion by partitioning the energy released between [V_2_O]^+^ and CO. To distinguish possible reactions of the charged particles with omnipresent background gases from those of the selected substrate, spectra with argon were recorded as well. As shown in Figure 1 a,f side reactions with background gases like trace amounts of oxygen are limited to the generation of small quantities of products typical for reactions of [V_2_]^+^ with O_2_,9e which are more pronounced in [V_2_O]^+^, since O_2_ has been added to the He buffer gas.

The rate constants *k*_4_([V_2_]^+^/CO_2_) and *k*_5_([V_2_O]^+^/CO_2_) for reactions shown in Equations (2) and (3) in Scheme 1 were measured and they amount to 3.8×10^−12^ (efficiency *φ*=0.5 %) and 8.8×10^−10^ cm^3^ molecule^−1^ s^−1^ (*φ*=1.15), respectively; these numbers translate to a ratio of *k*_5_/*k*_4_>230. Owing to an uncertainty in the determination of the absolute pressure of CO_2_, an error of ±30 % is associated with the rate measurements.5b For the relative rate constants, however, the error is much smaller, typically around ±5 %.

The considerable reactivity difference of [V_2_]^+^ versus [V_2_O]^+^ is unexpected and counter‐intuitive on the ground that the reactions in Equations (2) and (3) constitute redox processes in which electrons are transferred from the metal‐containing cluster ions to the CO_2_ molecule. Therefore, one would expect that [V_2_]^+^ reduces CO_2_ to CO more readily than the already oxidized and electron‐depleted [V_2_O]^+^. Consequently, *k*_4_ should be larger than *k*_5_; this is not the case. Why does the reactivity of [V_2_]^+^ and [V_2_O]^+^ not reflect their respective oxidation states? Further, does [V_2_O]^+^ react mechanistically the same way with CO_2_ as [V_2_]^+^? These questions have been addressed by density functional theory (DFT) calculations.

Earlier theoretical work had already shown that a description of vanadium dimers constitutes a notoriously difficult case to be dealt with by DFT calculations.^7^ This dilemma holds true as well for [V_2_O_2_]^+^.^8^ While for [V_2_O_2_]^+^ the gas‐phase IR spectrum suggests a planar, four‐membered V‐O‐V‐O ring structure of *C*_s_ symmetry,8b an unambiguous assignment of the electronic ground state (^2^A′ or ^6^A′) remained unresolved at the DFT levels employed.8b, 8c The problems are compounded by the observation that the gas‐phase chemistry of vanadium ions^9^ is often marred by a two‐ or multi‐state reactivity scenario.^10^ As to the bare [V_2_]^+^ cluster, due to its multireference character^7^, ^11^ we applied the ZORA‐NEVPT2(9e,12o)/ZORA‐def2‐QZVPP Scheme (Table S1 in the Supporting Information).^12^ According to these high‐level calculations, the ground state of [V_2_]^+^ corresponds to ^4^Σ_g_^−^. This assignment agrees well with experimental data and other, less sophisticated calculations.^13^ The lowest excited state of [V_2_]^+^ is a doublet (^2^Δ) and exceeds the ground state by only about 3 kJ mol^−1^ (Table S2). Therefore, it is very likely that under the given experimental conditions a mixture of two electronic states, ^2,4^[V_2_]^+^, is present, in which the quartet may dominate. We mention in passing that low‐energy excited states of the neutral V_2_ dimer were identified by Hübner and Himmel using matrix isolation spectroscopy.^11^ To construct a simplified potential energy surface (PES) for the [V_2_]^+^/[V_2_O]^+^/CO_2_ systems, extensive quantum chemical calculations were performed at the ZORA‐M06L/ZORA‐def2‐QZVPP//M06L/def2‐TZVP level of theory. Electronic structure calculations using the multi‐reference ZORA‐NEVPT2 scheme are not feasible technically. Rather, the M06L functional was chosen based on the exhaustive testing carried out by Truhlar and co‐workers.^7^ This functional performed best also among all the density functionals we benchmarked (see Tables S2, S6 and S7). In addition, M06L delivers fairly good results for the electronic states of [V_2_O]^+^ and [V_2_O_2_]^+^ (see Tables S3 and S4).

Figure 2 displays the PESs of the most favorable pathways for the reduction of CO_2_ with vanadium ions having doublet spin multiplicity as well as selected structural parameters of stationary points along the reaction coordinate. This particular spin state was chosen on the ground that along the whole transformation [V_2_]^+^ → [V_2_O_2_]^+^ the spin multiplicity was conserved. It should be mentioned at this point, however, that because of the strongly correlated electrons, the energetic and geometric properties of some stationary points with different spin multiplicities are located fairly close to each other. Therefore, it is quite possible that there is a superposition of the doublet with the quartet state and vice versa, facilitating a multi‐state reactivity scenario pending on the efficiency of spin‐orbit coupling (see below for a further comment).^10^ However, our aim is not to provide a quantitatively correct description of the whole PESs; rather, we aim at offering a qualitative understanding of the amazing reactivity behavior in the [V_2_]^+^/[V_2_O]^+^/CO_2_ systems. As the reactivity pattern proved to be quite complex, herein we limit ourselves to the main results. For further details, see Figures S2 and S3.

**Figure 2:**
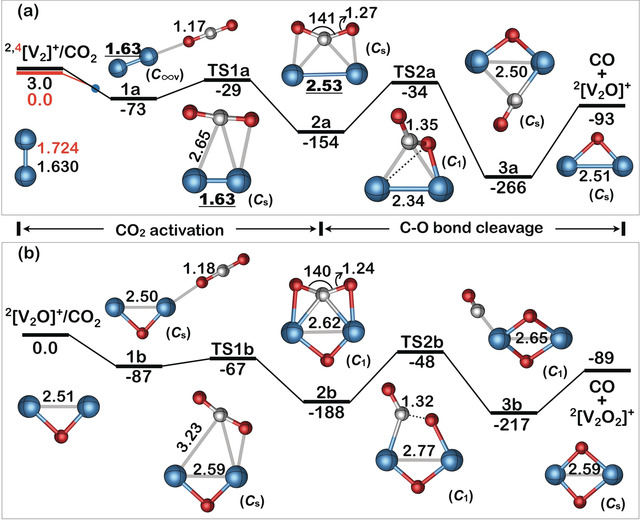
Schematic potential energy surfaces (Δ*H*_298K_ in kJ mol^−1^) obtained at the ZORA‐M06L/ZORA‐def2‐QZVPP//M06L/def2‐TZVP level of theory for the reactions of a) ^2^[V_2_]^+^ and b) ^2^[V_2_O]^+^ with CO_2_, respectively. Key structures with selected geometric parameters and symmetries are also provided. Bond lengths [Å]; angles [°]. The relative energies of ^2,4^[V_2_]^+^ were obtained at the ZORA‐NEVPT2(9e,12o)/ZORA‐def2‐QZVPP level of theory. For details, see the Supporting Information.

As displayed in Figure 2 a, at the doublet surface the exothermic OAT^14^ in the system [V_2_]^+^/CO_2_ commences with the barrier‐free formation of an encounter complex **1 a** by coordinating the incoming ligand to only one of the two vanadium atoms in an end‐on (*η* ^1^) mode. Subsequently, intermediate **2 a** is generated by surmounting transition state **TS1 a**, which is accompanied by a substantial elongation of the V−V bond from about 1.6 Å to 2.5 Å; this bond length does not change that much further in the remaining steps. In contrast, significant structural reorganization happens for CO_2_ at these stages. The initially linear substrate gets bent with an O‐C‐O angle of 141° in **2 a**, and the C−O bond length increases from 1.17 to 1.27 Å; clearly, CO_2_ is already markedly activated in this initial step. Next, the complete scission of one C−O bond of the CO_2_ moiety occurs along the route **2 a** → **TS2 a** → **3 a**. The reaction is completed by the release of CO to generate ^2^[V_2_O]^+^. The energetically submerged transition state **TS1 a**, as part of the reduction sequence of the first CO_2_ molecule, is with 29 kJ mol^−1^ located below the entrance asymptote and represents the rate‐limiting step of the entire reaction.

As to the exothermic reaction of [V_2_O]^+^ with CO_2_ and as displayed in Figure 2 b, ^2^[V_2_O]^+^ is approached by carbon dioxide as well, to then also form an *η* ^1^ encounter complex **1 b**; subsequently, this complex has sufficient internal energy to easily surmount transition state **TS1 b** and generate **2 b**. Next, the complete cleavage of the activated C−O bond takes place along the sequence **2 b** → **TS2 b** → **3 b**, and release of CO from **3 b** provides ^2^[V_2_O_2_]^+^. In contrast to the [V_2_]^+^/CO_2_ couple, here it is transition state **TS2 b** (−48 kJ mol^−1^) in which the C−O bond of the CO_2_ molecule is broken and which represents the rate‐limiting step in the reduction of CO_2_ to CO.

At first glance, the reductions of the two CO_2_ molecules by [V_2_]^+^ and [V_2_O]^+^ do not seem to differ too much in terms of structural features of the intermediates. So, why do they exhibit quite pronounced reactivity differences? As will be shown, these features can be attributed to both intrinsic structural and electronic properties. As suggested by a reviewer, one reason may be due to the spin situation. For the [V_2_O]^+^/CO_2_ couple the OAT is both exothermic and spin‐allowed (Figure 2 b); the whole reaction is confined to the doublet surface. This must not necessarily be the case for the [V_2_]^+^/CO_2_ system. While for electronically excited ^2^[V_2_]^+^ the reaction with CO_2_ is also both exothermic and spin‐allowed (Figure 2 a), for the ground state ^4^[V_2_]^+^ a quartet → doublet spin change has to occur along the overall exothermic reaction coordinate (Table S5). If this electronic reorganization is inefficient, as has been reported for the analogous exothermic [V]^+^/CO_2_ conversion,9f as well as for the [V]^+^/N_2_O couple,9a the reaction of [V_2_]^+^ with CO_2_ will be slowed down as well. Further, while thermochemical arguments on the *endo*‐ versus *exo*thermicity of a reaction,^7^ based only on DFT calculations, may be considered with caution, the insensitivity of the [V_2_]^+^/CO_2_ reactivity to intentional excitation of the [V_2_]^+^ projectile, strongly suggests that process (2) is exothermic and that its much reduced reactivity is not due to a thermodynamic impediment.

Indeed, there are other factors to be considered to explain the much higher reactivity of the [V_2_O]^+^/CO_2_ couple. Let us begin by looking at the charge distribution in the ionic actors. According to Figure 3 a, the reactive vanadium atoms in ^2^[V_2_O]^+^ carry more positive charge as compared to ^2^[V_2_]^+^. It is therefore expected that [V_2_O]^+^ will act as a worse electron donor for the reduction of CO_2_ compared to [V_2_]^+^.^15^ However, this is not the whole truth. In fact, the presence of the bridging oxygen atom in [V_2_O]^+^ actually plays a supportive role in the CO_2_ activation which overcompensates the unfavorable charge situation.

**Figure 3:**
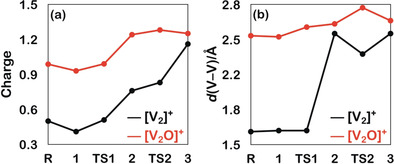
a) NBO‐calculated charges at the vanadium atoms coordinated to CO_2_ and b) development of the V−V bond length [Å] in the reactions of ^2^[V_2_]^+^ and ^2^[V_2_O]^+^ with CO_2_.

First, the presence of the oxygen atom induces a structural rearrangement of the active site, thus, providing a “preorganized” structure, which reduces substantially the energy demand to reach transition state **TS1 b**; this effect is well‐known from enzymatic processes.^16^ The calculated Wiberg Bond Index (WBI)^17^ of 4.5 indicates a rather strong and short V−V bond for [V_2_]^+^ (1.63 Å); a much smaller value of the WBI of 1.2 and an elongated V−V bond of 2.53 Å holds true for **2 a** (Figure 2 and Figure 3 b). As a consequence, stretching of the V−V bond in [V_2_]^+^ in the step **1 a** → **2 a** is quite energy‐demanding (Figure 2 a). In contrast, the corresponding transformation **1 b** to **2 b** in [V_2_O]^+^ requires much less energy as the V−V bond is already sufficiently elongated due to the presence of the oxygen bridge. Thus, [V_2_O]^+^ possesses a “prepared” structure which greatly enhances the reduction of CO_2_. In the remaining stages of the reaction there is no further dramatic change in the properties of the V−V bond, as indicated by the rather small oscillations of the WBI's around a value of 1. The moderate movements of the two vanadium atoms relative to each other do not require that much energy; rather, it is this structural change of the “active” site which assists in lowering **TS1 b** as compared with **TS1 a**.

There is yet another aspect to be considered. In ^2^[V_2_O]^+^ the presence of an oxygen atom raises the energy level of the electron‐donating d‐orbitals. As shown in Figure 4, in **TS1 a** of [V_2_]^+^ the overlap between the lobes of the doubly‐occupied πd_*xz*_/d_*xz*_‐orbital of the metal center and the vacant π*‐orbital of the carbon atom of CO_2_ has yet to be established. In contrast, for the early transition state **TS1 b**, the doubly‐occupied πd_*xz*_/d_*xz*_‐orbital is already delocalized quite extensively to the carbon atom of CO_2_, and the root cause for this difference can be traced back to orbital energy differences. The introduction of an oxygen bridge raises the πd_*xz*_/d_*xz*_‐orbital energy from −10.5 eV in [V_2_]^+^ to −9.5 eV in [V_2_O]^+^; this greatly eases the transfer of the d‐electrons into the empty π*‐orbital of CO_2_ thus favoring the reduction of [V_2_O]^+^.

**Figure 4:**
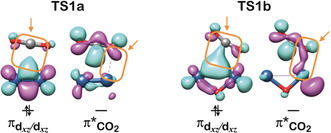
The frontier orbitals of the transition states of **TS1 a**,**b** from the doublet surface of the CO_2_ activation. Arrows and rounded rectangles indicate the overlap regions between the doubly occupied π‐electron‐donating orbital of the metals and the lowest empty orbital of CO_2_.

As shown repeatedly,^16^, ^18^ the presence or absence of “prepared states” are crucial in controlling the structural, electronic and energetic features of transition states. While there exist quite a number of examples for this scenario in the area of gas‐phase C−H bond activation,^19^ to our knowledge the present study describes for the first time this effect in the context of CO_2_ activation.

In conclusion, in the present experimental/computational work we report and explain quite a few unexpected observations:

1. A fleeting [V_2_O]^+^ intermediate is involved in the multistep oxidation of [V_2_]^+^ by CO_2_.
2. The counter‐intuitive oxidation behavior of [V_2_]^+^ versus [V_2_O]^+^ and the quite enormous reactivity differences of the two cluster ions have been traced back to the supportive role of the oxygen bridge in [V_2_O]^+^. This cluster oxide, in contrast to bare [V_2_]^+^, exhibits structural and electronic features which aid in the activation of CO_2_. While thermochemical aspects are not likely to cause the relative “inertness” of [V_2_]^+^, an inefficient quartet → doublet spin change in the [V_2_]^+^/CO_2_ couple may well matter in slowing down its reactivity.

## Conflict of interest

The authors declare no conflict of interest.

## Supporting information

As a service to our authors and readers, this journal provides supporting information supplied by the authors. Such materials are peer reviewed and may be re‐organized for online delivery, but are not copy‐edited or typeset. Technical support issues arising from supporting information (other than missing files) should be addressed to the authors.

Supplementary

## References

[1a] R. Huisgen , The Adventure Playground of Mechanisms and Novel Reactions (Profiles, Pathways, and Dreams), American Chemical Society, Washington DC, 1994;

[1b] R. Huisgen , Angew. Chem. Int. Ed. Engl. 1968, 7, 321–328;

[1c] R. Huisgen , J. Org. Chem. 1968, 33, 2291–2297;

[1d] R. Huisgen , Angew. Chem. Int. Ed. Engl. 1963, 2, 633–645;

[2] For an Essay on Huisgen's legacy, see:

[2a] K. N. Houk , H.-U. Reissig , Chem 2019, 5, 2499–2505; Also, see:

[2b] V. V. Rostovtsev , L. G. Green , V. V. Fokin , K. B. Sharpless , Angew. Chem. Int. Ed. 2002, 41, 2596–2599; 10.1002/1521-3773(20020715)41:14<2596::AID-ANIE2596>3.0.CO;2-4 12203546

[3a] J. Schwarz , C. Heinemann , D. Schröder , H. Schwarz , J. Hrušák , Helv. Chim. Acta 1996, 79, 1–5;

[3b] J. Schwarz , D. Schröder , H. Schwarz , C. Heinemann , J. Hrušák , Helv. Chim. Acta 1996, 79, 1110–1120.

[4] For a Review on gas-phase aspects, see: H. Schwarz , Coord. Chem. Rev. 2017, 334, 112–123.

[5a] M. Engeser , T. Weiske , D. Schröder , H. Schwarz , J. Phys. Chem. A 2003, 107, 2855–2859;

[5b] D. Schröder , H. Schwarz , D. E. Clemmer , Y. Chen , P. B. Armentrout , V. I. Baranov , D. K. Böhme , Int. J. Mass Spectrom. 1997, 161, 175–191;

[5c] K. Eller , H. Schwarz , Int. J. Mass Spectrom. 1989, 93, 243–257.

[6a] G. Kummerlöwe , M. K. Beyer , Int. J. Mass Spectrom. 2005, 244, 84–90;

[6b] T. Su , M. T. Bowers , J. Chem. Phys. 1973, 58, 3027–3037;

[6c] M. T. Bowers , J. B. Laudenslager , J. Chem. Phys. 1972, 56, 4711–4712.

[7] W. Zhang , D. G. Truhlar , M. Tang , J. Chem. Theory Comput. 2014, 10, 2399–2409. 26580760 10.1021/ct500296a

[8a] R.-Z. Li , H.-G. Xu , G.-J. Cao , Y.-C. Zhao , W.-J. Zheng , Chin. J. Chem. Phys. 2011, 24, 572–579;

[8b] K. R. Asmis , G. Meijer , M. Brümmer , C. Kaposta , G. Santambrogio , L. Wöste , J. Sauer , J. Chem. Phys. 2004, 120, 6461–6470; 15267535 10.1063/1.1650833

[8c] M. Calatayud , J. Andrés , A. Beltrán , J. Phys. Chem. A 2001, 105, 9760–9775.

[9] For some typical examples, see:

[9a] S. G. Ard , B. C. Sweeny , D. C. McDonald , A. A. Viggiano , N. S. Shuman , J. Phys. Chem. A 2020, 124, 30–38; 31790258 10.1021/acs.jpca.9b09235

[9b] Y. C. Chang , Y. Xu , C.-Y. Ng , Phys. Chem. Chem. Phys. 2019, 21, 6868–6877; 30887995 10.1039/c9cp00575g

[9c] H. Zhang , H. Wu , L. Geng , Y. Jia , M. Yang , Z. Luo , Phys. Chem. Chem. Phys. 2019, 21, 11234–11241, and references therein; 31099360 10.1039/c9cp01192g

[9d] G. K. Koyanagi , D. K. Bohme , J. Phys. Chem. A 2006, 110, 1232–1241; 16435784 10.1021/jp0526602

[9e] C. Rue , P. B. Armentrout , I. Kretzschmar , D. Schröder , J. N. Harvey , H. Schwarz , J. Chem. Phys. 1999, 110, 7858–7870;

[9f] J. Xu , M. T. Rodgers , J. B. Griffin , P. B. Armentrout , J. Chem. Phys. 1998, 108, 9339–9350;

[9g] M. R. Sievers , P. B. Armentrout , J. Chem. Phys. 1995, 102, 754–762;

[9h] M. M. Kappes , R. H. Staley , J. Phys. Chem. 1981, 85, 942–944.

[10] For a selection of Reviews, see:

[10a] S. Shaik , Isr. J. Chem. 2020, DOI: 10.1002/ijch.202000002;

[10b] D. Ricciarelli , L. Belpassi , J. N. Harvey , P. Belanzoni , Chem. Eur. J. 2020, 26, 3080–3089; 31846105 10.1002/chem.201904314

[10c] D. Mandal , D. Mallick , S. Shaik , Acc. Chem. Res. 2018, 51, 107–117; 29297671 10.1021/acs.accounts.7b00442

[10d] T. Takayanagi , T. Nakatomi , J. Comput. Chem. 2018, 39, 1319–1326; 29504140 10.1002/jcc.25202

[10e] J. N. Harvey , Wiley Interdiscip. Rev.: Comput. Mol. Sci. 2014, 4, 1–14;

[10f] S. Shaik , H. Hirao , D. Kumar , Acc. Chem. Res. 2007, 40, 532–542; 17488054 10.1021/ar600042c

[10g] F. Neese , T. Petrenko , D. Ganyushin , G. Olbrich , Coord. Chem. Rev. 2007, 251, 288–327;

[10h] J. N. Harvey , Phys. Chem. Chem. Phys. 2007, 9, 331–343; 17199148 10.1039/b614390c

[10i] S. Shaik , D. Kumar , S. P. de Visser , A. Altun , W. Thiel , Chem. Rev. 2005, 105, 2279–2328; 15941215 10.1021/cr030722j

[10j] H. Schwarz , Int. J. Mass Spectrom. 2004, 237, 75–105;

[10k] R. Poli , J. N. Harvey , Chem. Soc. Rev. 2003, 32, 1–8; 12596540 10.1039/b200675h

[10l] S. Shaik , S. P. de Visser , F. Ogliaro , H. Schwarz , D. Schröder , Curr. Opin. Chem. Biol. 2002, 6, 556–567; 12413538 10.1016/s1367-5931(02)00363-0

[10m] D. Schröder , S. S. Shaik , H. Schwarz , Acc. Chem. Res. 2000, 33, 139–145; 10727203 10.1021/ar990028j

[10n] S. Shaik , M. Filatov , D. Schröder , H. Schwarz , Chem. Eur. J. 1998, 4, 193–199;

[10o] P. B. Armentrout , Science 1991, 251, 175–179; 17836946 10.1126/science.251.4990.175

[10p] P. B. Armentrout , Annu. Rev. Phys. Chem. 1990, 41, 313–344.

[11] O. Hübner , H.-J. Himmel , Phys. Chem. Chem. Phys. 2016, 18, 14667–14677. 27182729 10.1039/c6cp00835f

[12a] C. Angeli , R. Cimiraglia , J.-P. Malrieu , J. Chem. Phys. 2002, 117, 9138–9153;

[12b] C. Angeli , R. Cimiraglia , S. Evangelisti , T. Leininger , J. P. Malrieu , J. Chem. Phys. 2001, 114, 10252–10264;

[12c] C. Angeli , R. Cimiraglia , J.-P. Malrieu , Chem. Phys. Lett. 2001, 350, 297–305.

[13a] D. S. Yang , A. M. James , D. M. Rayner , P. A. Hackett , J. Chem. Phys. 1995, 102, 3129–3134;

[13b] A. M. James , P. Kowalczyk , E. Langlois , M. D. Campbell , A. Ogawa , B. Simard , J. Chem. Phys. 1994, 101, 4485–4495;

[13c] L. M. Russon , S. A. Heidecke , M. K. Birke , J. Conceicao , M. D. Morse , P. B. Armentrout , J. Chem. Phys. 1994, 100, 4747–4755;

[13d] B. Simard , A. James , P. Kowalczyk , R. Fournier , P. Hackett , Proc. SPIE-Int. Soc. Opt. Eng. 1994, 2124, 376–387.

[14a] Given the well-established problems with DFT-based energetics (see, for example, Ref. [7], a reviewer has raised the possibility that the reduced reactivity of [V_2_]^+^ towards CO_2_ is due to an unfavorable thermochemistry, that is, the formation of [V_2_O]^+^ is either thermoneutral or slightly endothermic. This option has been tested by intentional kinetic excitation of [V_2_]^+^ prior to its reaction with CO_2_ (for technical details, see: M. Beyer , V. E. Bondybey , Rapid Commun. Mass Spectrom. 1997, 11, 1588–1591): Up to a time regime of 40 μs, neither the amount of the precursor ion [V_2_]^+^ nor that of the product ion [V_2_O_2_]^+^ are affected by kinetically exciting the [V_2_]^+^ projectile. At much longer excitation time, the intensity of the [V_2_O_2_]^+^ signal goes through a maximum while that of [V_2_]^+^ gets continuously weaker due to the ions’ ejection from the ICR cell. This behavior corresponds to an exothermic reactivity scenario of the [V_2_]^+^/CO_2_ couple and is further experimentally supported by the fact that at room temperature no formation of [V_2_]^+^ is observed in the reaction of [V_2_O]^+^ with CO, which represents the reversal of [Eq. (2)]. Since there is no oxygen exchange when [V_2_O]^+^ is exposed to ^13^C^18^O, the energy of **TS2a** in Figure 2a must exceed that of the product pair [V_2_O]^+^/CO, confirming the results of the calculations.

[14b] According to previous experimental work,^[9d,g,h]^ the reaction of ground-state ^5^[V]^+^ with CO_2_ to form ^3^[VO]^+^ and CO is exothermic and spin-forbidden. For this process our M06L/DEF2-TZVP calculations predict a barrier of 74 kJ mol^−1^ above the entrance channel; this explains why this redox process has not been observed using SIFT or ICR.^[9d,h]^

[15] T. Fan , X. Chen , Z. Lin , Chem. Commun. 2012, 48, 10808–10828. 10.1039/c2cc34542k 22991691

[16] A. J. T. Smith , R. Müller , M. D. Toscano , P. Kast , H. W. Hellinga , D. Hilvert , K. N. Houk , J. Am. Chem. Soc. 2008, 130, 15361–15373. 18939839 10.1021/ja803213p PMC2728765

[17] K. B. Wiberg , Tetrahedron 1968, 24, 1083–1096.

[18a] W. Lai , C. Li , H. Chen , S. Shaik , Angew. Chem. Int. Ed. 2012, 51, 5556–5578; 10.1002/anie.201108398 22566272

[18b] S. Ye , F. Neese , Proc. Natl. Acad. Sci. USA 2011, 108, 1228–1233; 21220293 10.1073/pnas.1008411108 PMC3029731

[18c] S. S. Shaik , J. Am. Chem. Soc. 1981, 103, 3692–3701.

[19a] C. Geng , J. Li , T. Weiske , H. Schwarz , Chem. Eur. J. 2019, 25, 12940–12945; 31268193 10.1002/chem.201902572 PMC6852486

[19b] J. Li , X.-N. Wu , M. Schlangen , S. Zhou , P. González-Navarrete , S. Tang , H. Schwarz , Angew. Chem. Int. Ed. 2015, 54, 5074–5078; 10.1002/anie.201412441 25728585

[19c] H. Schwarz , Isr. J. Chem. 2014, 54, 1413–1431.

