# Supplementary material for: Counter‐Intuitive Gas‐Phase Reactivities of [V2]+ and [V2O]+ towards CO2 Reduction: Insight from Electronic Structure Calculations
Source: Angew Chem Int Ed Engl. 2020 Mar 25;59(30):12308–14. doi: 10.1002/anie.202001223 (PMC7383893; doi:10.1002/anie.202001223)
Supplement: Supplementary file 1 — Supplementary [file ANIE-59-12308-s001.pdf]

Angewandte Chemie
Eine Zeitschrift der Gesellschaft Deutscher Chemiker

Supporting Information

Counter-Intuitive Gas-phase Reactivities of $[V_2]^+$ and $[V_2O]^+$ towards CO_2 Reduction: Insight from Electronic Structure Calculations

Jilai Li, Caiyun Geng, Thomas Weiske, and Helmut Schwarz**

anie_202001223_sm_miscellaneous_information.pdf

Table of Contents

1. Experimental Details 2

2. Computational Details 2

3. Figures 4

4. Tables 7

5. Coordinates of transition state structures in Figure 2 13

6. References 14

1. Experimental Details

The ion/molecule reactions were performed in a Spectrospin CMS 47X Fourier transform ion cyclotron resonance (FT-ICR) mass spectrometer equipped with an external ion source as described elsewhere.[1] Briefly, $[V_2]^+$ was generated by laser ablation of a vanadium target using a Nd:YAG laser operating at 532 nm; helium mixed with trace amount of N_2 served as a cooling and carrier gas. It is important that the helium pipe has been baked beforehand to remove impurities on the inner walls of the feeding pipes and thus significantly improve the production of $[V_2]^+$. Using a series of ion lenses, the ions were transferred into the ICR cell, which is positioned in the bore of a 7.05 T superconducting magnet. After thermalization by about 1×10^5 collisions with pulsed-in argon, the reactions of mass-selected $[V_2]^+$ were studied by introducing isotopologues of carbon dioxide (CO_2 and $C^{18}O_2$) and a 1 : 1 mixture of CO_2 and $C^{18}O_2$ via leak valves at stationary pressures. In the $[V_2O]^+/CO_2$ experiments, trace amounts of O_2 were mixed with helium for the generation of $[V_2O]^+$. A temperature of 298 K was assumed for the thermalized clusters.[1]

The rate constants have been determined following the detailed protocol documented in the PhD Thesis of K. Koszinowski.[2] Typically, the pressures are determined with an uncalibrated Bayard-Alpard ion gauge whose reading differs depending on the kind of the gas. As the concentration of the ionic reactant A^+ is small compared to the neutral substrate B, a pseudo first-order reaction can be assumed as a good approximation,

$$d[A^+]/dt = -k[A^+][B] \cong -k_{obs}[A^+], \text{ with } -k_{obs} = -k[B]$$

here k is the true bimolecular and k_{obs} represents the apparent pseudo-unimolecular rate constant. Recording a time-dependent profile of the natural logarithm of the normalized intensity of the educt ions delivers the decline of the reactant ions whose negative slope corresponds to k_{obs}. For a general procedure to determine reaction-rate constants of ions with neutrals in the diluted gas phase, see reference.[3]

2. Computational Details

The calculations of the electronic structures were performed with Gaussian and ORCA.[4]

Quite elaborate multireference (MR) calculations were conducted to determine the relative energies of low-lying electronic states of $[V_2]^+$. To treat dynamic correlation without the problems of intruder states or level shifts,[5] *n*-electron valence perturbation theory (NEVPT2)[6] in conjunction with the ZORA-def2-QZVPP basis set,[7] as implemented in ORCA, was employed to optimize the geometries. Active space (9e,12o) has been considered in these calculations. The energetic information is given in Table S1; for the selection of the active space, see Figure S1.

We also conducted density functional theory (DFT) calculations by using various functionals, such as M06L,[8] HFS,[9] BP86,[10] TPSS,[11] VWN5,[12] BLYP,[10b,13] OLYP,[14] XLYP,[15] PW91,[16] RPBE,[17] PWP,[10,16] B3LYP,[10b,13,18] O3LYP,[19] X3LYP,[15] PBE0,[20] TPSSH,[21] M06,[22] CAM-B3LYP,[23] B2PLYP,[24] and SCAN[25] in combination with the ZORA-def2-TZVP[7] basis set for the low-lying electronic states of $[V_2]^+$, $[V_2O]^+$, $[V_2O_2]^+$ as well as for the process $[V_2]^+ + CO_2 \rightarrow [V_2O]^+ + CO$ for different spin states. The relative energies are shown in Tables S2-S5.

We used the M06L[8] density functional in combination with the def2-TZVP[7a,b] basis set for the structural optimization to model the potential energy surfaces of the reactions. Harmonic vibrational frequencies were computed to verify the nature of the stationary points. The minimum structures reported in this paper show only positive eigenvalues of the Hessian matrix, whereas the transition states (TSs) have only one negative eigenvalue. Intrinsic reaction coordinate[26] calculations were also performed to confirm that the transition states correlate with designated intermediates. The thermodynamic functions (ΔH) were estimated within the ideal gas, rigid-rotor, and harmonic oscillator approximations at 298 K and 1 atm.

As commonly accepted, the geometries of molecular structures are less dependent on the level of theory than the energies thus obtained. For further energetic refinements, single-point energy (SPE) calculations at the ZORA-M06L/ZORA-def2-QZVPP level of theory were performed for the structures as optimized by the aid of the M06L functional.

3. Figures

Figure S1. Selected active spaces considered in the ZORA-NEVPT2(9e,12o)//ZORA-def2-QZVPP calculations for $^{2}[V_2]^+$, $^{4}[V_2]^+$, $^{6}[V_2]^+$, and $^{8}[V_2]^+$, respectively. Natural orbital partial occupation numbers are given.

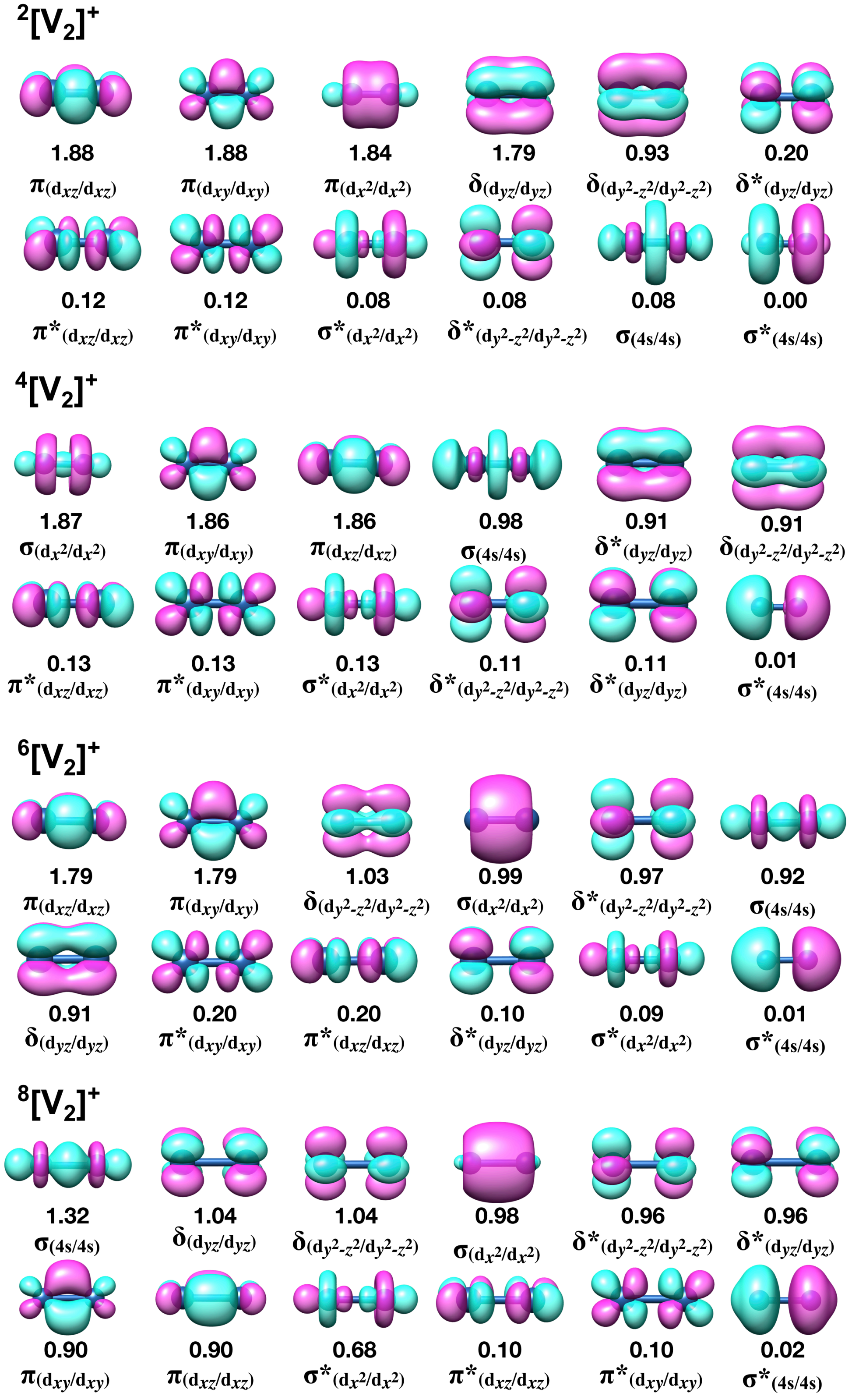

Figure S2. Potential energy surfaces (ΔH_{298K} in kJ mol^{-1}) as obtained at the ZORA-M06L/ZORA-def2-QZVPP//M06L/def2-TZVP level of theory for the reactions of (a) $[V_2]^+$ and (b) $[V_2O]^+$ with CO_2. Color code: doublet, black; quartet, blue; sextet, red; octet, magenta.

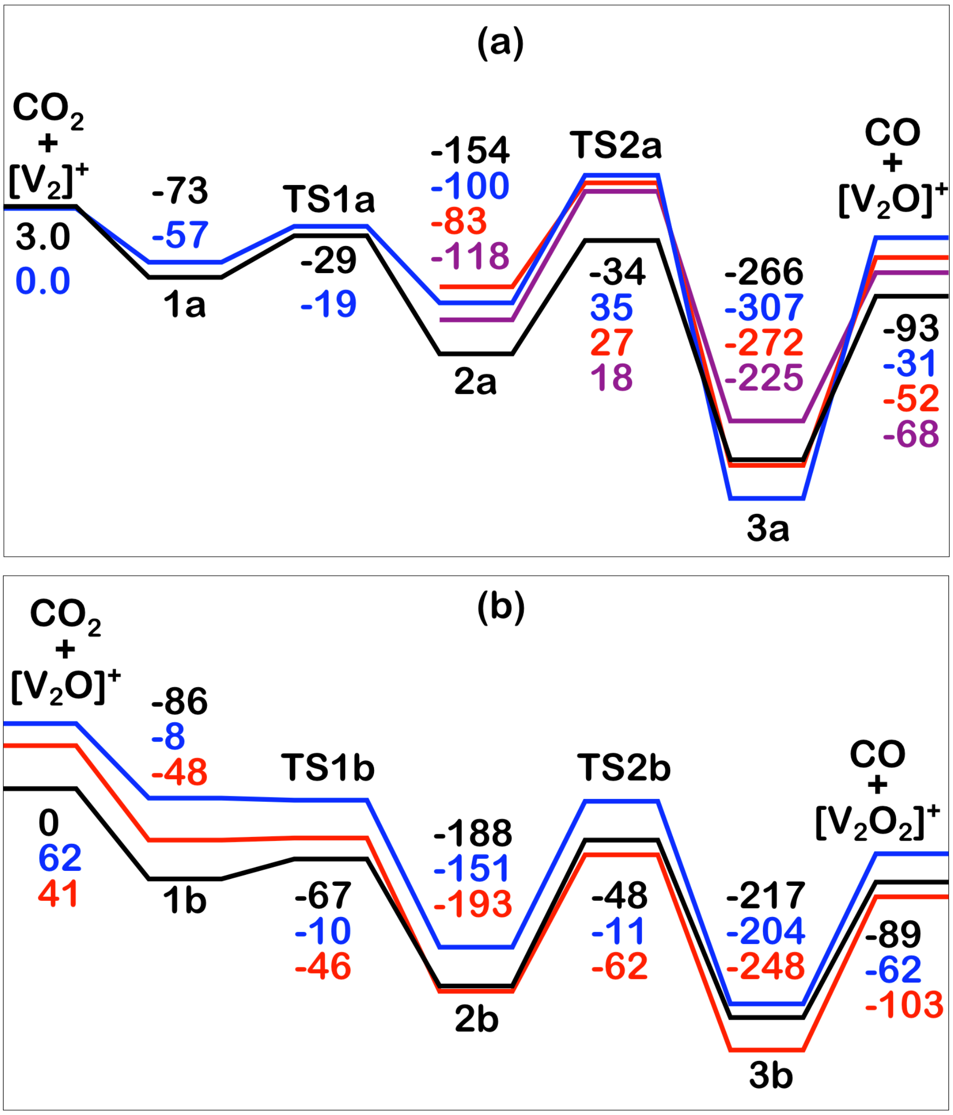

Figure S3. Key structures with selected geometric parameters for the reactions of (a) $[V_2]^+$, and (b) $[V_2O]^+$ with CO_2 as obtained at the M06L/def2-TZVP level of theory. Bond lengths are given in Å. Charges are omitted for the sake of clarity. Color code: doublet, black; quartet, blue; sextet, red; octet, magenta.

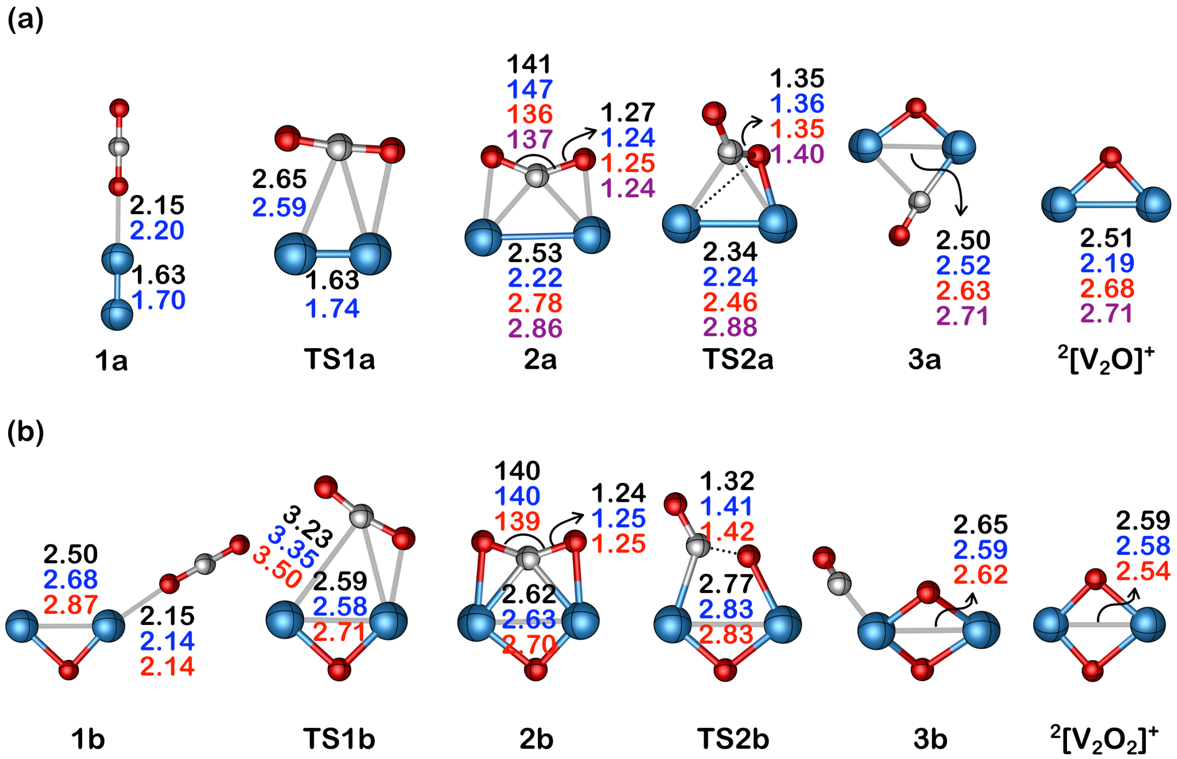

4. Tables

Table S1. Relative energies of different states of $[V_2]^+$ (ΔH in kJ mol^{-1}) as obtained at the ZORA-NEVPT2/ZORA-def2-QZVPP level of theory. Bond distances (R_e) are given in Å.

	ΔH	R_e
$^2[V_2]^+$	3	1.630
$^4[V_2]^+$	0	1.724
$^6[V_2]^+$	123	1.906
$^8[V_2]^+$	184	2.333

Table S2. Relative energies (ΔE in kJ mol^{-1}) of $^2[V_2]^+$ and $^4[V_2]^+$ as calculated by various density functionals using the ZORA-def2-TZVP basis set.

	$^2[V_2]^+$	$^4[V_2]^+$
M06L	1	0
M06	3	0
CAM-B3LYP	-5	0
VWN5	8	0
O3LYP	12	0
OLYP	15	0
TPSS	16	0
TPSSH	18	0
PW91	24	0
BP86	25	0
PWP	26	0
SCAN	27	0
BLYP	29	0
RPBE	29	0
PBE0	29	0
XLYP	30	0
B3LYP	30	0
X3LYP	31	0
HFS	61	0
B2PLYP	127	0

Table S3. Relative energies (ΔE in kJ mol^{-1}) of two low-lying electronic states of $[V_2O]^+$ as calculated by various density functionals using the ZORA-def2-TZVP basis set.

	$^2[V_2O]^+$	$^8[V_2O]^+$
HFS	0	-51
SCAN	0	-19
PBE0	0	-14
OLYP	0	-9
O3LYP	0	-9
TPSSH	0	-6
RPBE	0	-4
BP86	0	-3
CAM-B3LYP	0	-3
B3LYP	0	-2
X3LYP	0	-2
PW91	0	1
B2PLYP	0	1
TPSS	0	3
M06L	0	6
M06	0	6
PWP	0	9
BLYP	0	17
XLYP	0	19
VWN5	0	96

Table S4. Relative energies (ΔE in kJ mol^{-1}) of three low-lying electronic states of $[V_2O_2]^+$ as calculated by various density functionals using the ZORA-def2-TZVP basis set.

	$^2[V_2O_2]^+$	$^4[V_2O_2]^+$	$^6[V_2O_2]^+$
HFS	0	6	-31
LDA	0	-18	-33
VWN5	0	-18	-33
O3LYP	0	35	-16
BP86	0	8	-14
PWP	0	5	-14
OLYP	0	12	-14
PW91	0	7	-13
M06L	0	24	-12
RPBE	0	11	-9
M06	0	54	-6
BLYP	0	11	-3
XLYP	0	11	-1
TPSSH	0	27	0
TPSS	0	14	1
SCAN	0	12	1
PBE0	0	37	2
B3LYP	0	33	3
X3LYP	0	33	3
CAM-B3LYP	0	34	5
B2PLYP	0	53	[a]

[a]) this structure did not converge.

Table S5. Reaction energies (ΔE in kJ mol^{-1}) for Eqs. (1)-(4) as calculated by various density functionals using the ZORA-def2-TZVP basis set.

$$^{2}[V_2]^{+} + CO_2 \rightarrow {}^{2}[V_2O]^{+} + CO \quad (1)$$
$$^{4}[V_2]^{+} + CO_2 \rightarrow {}^{2}[V_2O]^{+} + CO \quad (2)$$
$$^{4}[V_2]^{+} + CO_2 \rightarrow {}^{8}[V_2O]^{+} + CO \quad (3)$$
$$^{2}[V_2]^{+} + CO_2 \rightarrow {}^{8}[V_2O]^{+} + CO \quad (4)$$

	**Eq. 1**	**Eq. 2**	**Eq. 3**	**Eq. 4**
HFS	-73	-12	-64	-124
BP86	-38	-12	-15	-41
TPSS	-77	-61	-59	-75
VWN5	-17	-9	87	79
BLYP	-55	-27	-10	-38
OLYP	-19	-4	-13	-28
M06L	-94	-93	-88	-88
XLYP	-62	-32	-13	-42
PW91	-30	-7	-6	-30
RPBE	-56	-27	-31	-60
PWP	-34	-8	1	-25
B3LYP	-184	-154	-157	-186
O3LYP	-53	-41	-50	-62
X3LYP	-198	-167	-169	-199
PBE0	-212	-183	-198	-226
TPSSH	-140	-122	-128	-147
M06	-117	-114	-108	-111
CAM-B3LYP	-210	-215	-218	-213
B2PLYP	-91	35	37	-90
SCAN	-181	-153	-112	-139

Table S6. Experimental bond dissociation energies (kJ mol^{-1}) of various species.

		ΔE	(±)
**A:**	$V_2O^{+} \rightarrow V_2^{+} + {}^{3}O$	493[27]	29
		656[1a]	48
**B:**	$V_2O^{+} \rightarrow VO^{+} + V$	205[27]	39
		368[1a]	58
**C:**	$VO^{+} \rightarrow V^{+} + O$	578[27]	9.6
**D:**	$VO \rightarrow V + O$	625[28]	19
**E:**	$V_2^{+} \rightarrow V^{+} + V$	303[29]	0.2
**F:**	$VC^{+} \rightarrow V^{+} + C$	373[30]	14

Table S7. Calculated bond dissociation energies (kJ mol^{-1}) of various species **A** to **F** from Table 6 by using available functionals in combination with the def2-TZVP basis set implemented in ORCA 4.2. X columns refer to the error bar of the calculations. Numbers are missing (marked by "--") where calculations with a certain functional have not converged.

ΔE	A			B			C		D		E		F	
Exp	[493,656]	X[1a]	X[27]	[205,368]	X[1a]	X[27]	578	X	625	X	303	X	373	X
HFS	679	23	186	352	16	147	636	58	669	44	309	6	348	25
VWN	649	7	156	420	52	215	773	195	815	190	543	240	496	123
VWN3	728	72	235	509	141	304	790	212	831	206	571	268	518	145
PWLDA	644	12	151	416	48	211	773	195	815	190	545	242	498	125
BP86	610	46	117	341	27	136	643	65	694	69	374	71	403	30
BLYP	586	70	93	335	33	130	639	61	696	71	389	86	396	23
OLYP	587	69	94	309	59	104	615	37	668	43	337	34	370	3
XLYP	586	70	93	339	29	134	637	59	694	69	389	86	395	22
PW91	616	40	123	343	25	138	655	77	704	79	382	79	414	41
mPWPW	615	41	122	--	--	--	641	63	--	--	--	--	403	30
mPWLYP	593	63	100	349	19	144	650	72	707	82	407	104	405	32
PBE	615	41	122	339	29	134	654	76	702	77	378	75	414	41
RPBE	601	55	108	318	50	113	617	39	666	41	334	31	385	12
REVPBE	605	51	112	319	49	114	619	41	668	43	334	31	387	14
PWP	612	44	119	356	12	151	668	90	718	93	412	109	424	51
B1LYP	728	72	235	335	33	130	510	68	584	41	118	185	276	97
B3LYP	705	49	212	331	37	126	542	36	610	15	168	135	304	69
O3LYP	659	3	166	328	40	123	610	32	668	43	279	24	357	16
X3LYP	714	58	221	336	32	131	538	40	608	17	160	143	300	73
B1P	741	85	248	337	31	132	515	63	583	42	111	192	285	88
B3P	715	59	222	333	35	128	546	32	610	15	164	139	312	61
B3PW	718	62	225	323	45	118	536	42	598	27	141	162	303	70
PW1PW	748	92	255	--	--	--	521	57	--	--	--	--	290	83
mPW1PW	747	91	254	--	--	--	511	67	--	--	--	--	282	91
mPW1LYP	741	85	248	341	27	136	519	59	592	33	119	184	283	90
PBE0	748	92	255	333	35	128	521	57	586	39	105	198	291	82
PW6B95	732	76	239	--	--	--	526	52	--	--	--	--	292	81
BHANDHLYP	592	64	99	372	4	167	392	186	487	138	172	131	184	189
TPSS	617	39	124	351	17	146	604	26	662	37	338	35	391	18
TPSSH	673	17	180	344	24	139	555	23	617	8	225	78	344	29
TPSS0	744	88	251	351	17	146	485	93	556	69	92	211	275	98
M06L	645	11	152	362	6	157	577	1	629	4	294	9	385	12
M06	668	12	175	332	36	127	550	28	618	7	214	89	315	58
M062X	647	9	154	346	22	141	498	80	542	83	196	107	247	126
B97M-D3BJ	598	58	105	435	67	230	547	31	659	34	383	80	341	32
wB97	816	160	323	311	57	106	717	139	740	115	211	92	324	49

wB97X	701	45	208	316	52	111	537	41	585	40	153	150	295	78
wB97X-D3BJ	681	25	188	357	11	152	533	45	607	18	209	94	292	81
CAM-B3LYP	739	83	246	342	26	137	527	51	605	20	129	174	275	98
LC-BLYP	789	133	296	358	10	153	734	156	801	176	303	0	360	13
B2PLYP	--	--	--	282	86	77	587	9	650	25	--	--	375	2
mPW2PLYP	--	--	--	--	--	--	562	16	--	--	--	--	338	35
B2GP-PLYP	--	--	--	284	84	79	557	21	625	0	--	--	365	8
B2K-PLYP	--	--	--	--	--	--	538	40	609	16	--	--	360	13
B2T-PLYP	--	--	--	285	83	80	563	15	629	4	--	--	357	16
PWPB95	--	--	--	--	--	--	552	26	--	--	--	--	331	42
DSD-BLYP	--	--	--	276	92	71	566	12	638	13	--	--	419	46
DSD-PBEP86	--	--	--	--	--	--	551	27	622	3	--	--	231	142
wB2PLYP	--	--	--	--	--	--	539	39	613	12	--	--	270	103
wB2GP-PLYP	--	--	--	--	--	--	533	45	607	18	--	--	295	78

5. Coordinates of transition state structures in Figure 2

^{2}TS1a

V	-0.951303	0.792256	-0.002180
V	-0.793223	-0.834583	-0.002005
C	1.582742	0.028542	0.001329
O	1.707759	1.188046	0.001556
O	1.568171	-1.147519	0.001308

^{2}TS2a

V	1.146224	0.156944	0.489567
V	-0.605031	-1.251133	-0.170070
C	-0.812328	0.900990	0.110513
O	0.167447	1.126893	-0.787519
O	-1.825474	1.468125	0.356127

^{2}TS1b

V	-1.274767	-1.007444	-0.000225
V	-0.055712	1.283166	0.000151
O	-1.712102	0.783181	0.000102
C	1.920467	-0.561843	0.000000
O	2.129885	-1.692791	-0.000117
O	1.942474	0.629722	0.000136

^{2}TS2b

V	-0.531774	-0.341199	-0.715090
V	1.975781	-0.682864	0.415169
O	0.617672	-1.626314	-0.399191
C	0.187965	1.534035	-0.184041
O	-0.218279	2.638047	-0.359734
O	1.231668	1.089477	0.489323

6. References

[1] (a) M. Engeser, T. Weiske, D. Schröder, H. Schwarz, *J. Phys. Chem. A* **2003**, *107*, 2855–2859; (b) D. Schröder, H. Schwarz, D. E. Clemmer, Y. Chen, P. B. Armentrout, V. I. Baranov, D. K. Böhme, *Int. J. Mass Spectrom.* **1997**, *161*, 175–191; (c) K. Eller, H. Schwarz, *Int. J. Mass Spectrom.* **1989**, *93*, 243–257.

[2] K. Koszinowski, Gaseous Platinum Clusters - Versatile Models for Heterogeneous Catalysts, PhD Thesis, Technische Universität Berlin, D 83, **2003**.

[3] A. G. Marshall, S. E. Buttrill, *J. Chem. Phys.* **1970**, *52*, 2752–2759.

[4] (a) M. J. Frisch, G. W. Trucks, H. B. Schlegel, G. E. Scuseria, M. A. Robb, J. R. Cheeseman, G. Scalmani, V. Barone, B. Mennucci, G. A. Petersson, H. Nakatsuji, M. Caricato, X. Li, H. P. Hratchian, A. F. Izmaylov, J. Bloino, G. Zheng, J. L. Sonnenberg, M. Hada, M. Ehara, K. Toyota, R. Fukuda, J. Hasegawa, M. Ishida, T. Nakajima, Y. Honda, O. Kitao, H. Nakai, T. Vreven, J. J. A. Montgomery, J. E. Peralta, F. Ogliaro, M. Bearpark, J. J. Heyd, E. Brothers, K. N. Kudin, V. N. Staroverov, T. Keith, R. Kobayashi, J. Normand, K. Raghavachari, A. Rendell, J. C. Burant, S. S. Iyengar, J. Tomasi, M. Cossi, N. Rega, J. M. Millam, M. Klene, J. E. Knox, J. B. Cross, V. Bakken, C. Adamo, J. Jaramillo, R. Gomperts, R. E. Stratmann, O. Yazyev, A. J. Austin, R. Cammi, C. Pomelli, J. W. Ochterski, R. L. Martin, K. Morokuma, V. G. Zakrzewski, G. A. Voth, P. Salvador, J. J. Dannenberg, S. Dapprich, A. D. Daniels, O. Farkas, J. B. Foresman, J. V. Ortiz, J. Cioslowski, D. J. Fox, Gaussian 09, Revision D.01, Gaussian, Inc., Wallingford CT, **2013**; (b) F. Neese, *WIREs Comput. Mol. Sci.* **2018**, *8*, e1327.

[5] S. Guo, M. A. Watson, W. Hu, Q. Sun, G. K.-L. Chan, *J. Chem. Theory Comput.* **2016**, *12*, 1583-1591.

[6] C. Angeli, R. Cimiraglia, S. Evangelisti, T. Leininger, J. P. Malrieu, *J. Chem. Phys.* **2001**, *114*, 10252-10264.

[7] (a) F. Weigend, R. Ahlrichs, *Phys. Chem. Chem. Phys.* **2005**, *7*, 3297-3305; (b) F. Weigend, *Phys. Chem. Chem. Phys.* **2006**, *8*, 1057-1065; (c) D. A. Pantazis, X.-Y. Chen, C. R. Landis, F. Neese, *J. Chem. Theory Comput.* **2008**, *4*, 908-919.

[8] Y. Zhao, D. G. Truhlar, *J. Chem. Phys.* **2006**, *125*, 194101.

[9] (a) P. Hohenberg, W. Kohn, *Phys. Rev. B* **1964**, *136*, B864-B871; (b) W. Kohn, L. J. Sham, *Phys. Rev.* **1965**, *140*, A1133-A1138.

[10] (a) J. P. Perdew, *Phys. Rev. B* **1986**, *33*, 8822-8824; (b) A. D. Becke, *Phys. Rev. A* **1988**, *38*, 3098-3100.

[11] J. Tao, J. P. Perdew, V. N. Staroverov, G. E. Scuseria, *Phys. Rev. Lett.* **2003**, *91*, 146401.

[12] S. H. Vosko, L. Wilk, M. Nusair, *Can. J. Phys.* **1980**, *58*, 1200-1211.

[13] C. Lee, W. Yang, R. G. Parr, *Phys. Rev. B* **1988**, *37*, 785-789.

[14] (a) J. Baker, P. Pulay, *J. Comput. Chem.* **2003**, *24*, 1184-1191; (b) W.-M. Hoe, A. J. Cohen, N. C. Handy, *Chem. Phys. Lett.* **2001**, *341*, 319-328.

[15] X. Xu, W. A. Goddard III, *Proc. Natl. Acad. Sci. USA* **2004**, *101*, 2673-2677.

[16] (a) J. P. Perdew, J. A. Chevary, S. H. Vosko, K. A. Jackson, M. R. Pederson, D. J. Singh, C. Fiolhais, *Phys. Rev. B* **1992**, *46*, 6671–6687; (b) J. P. Perdew, K. Burke, Y. Wang, *Phys. Rev. B* **1996**, *54*, 16533–16539.

[17] B. Hammer, L. B. Hansen, J. K. Nørskov, *Phys. Rev. B* **1999**, *59*, 7413-7421.

[18] (a) A. D. Becke, *J. Chem. Phys.* **1993**, *98*, 5648-5652; (b) P. J. Stephens, F. J. Devlin, C. F. Chabalowski, M. J. Frisch, *J. Phys. Chem.* **1994**, *98*, 11623–11627.

[19] A. J. Cohen, N. C. Handy, *Mol. Phys.* **2001**, *99*, 607-615.

[20] C. Adamo, V. Barone, *J. Chem. Phys.* **1999**, *110*, 6158-6170.

[21] V. N. Staroverov, G. E. Scuseria, J. Tao, J. P. Perdew, *J. Chem. Phys.* **2003**, *119*, 12129-12137.

[22] Y. Zhao, D. Truhlar, *Theor. Chem. Acc.* **2008**, *120*, 215-241.

[23] T. Yanai, D. P. Tew, N. C. Handy, *Chem. Phys. Lett.* **2004**, *393*, 51-57.

[24] S. Grimme, *J. Chem. Phys.* **2006**, *124*, 034108.

[25] J. Sun, A. Ruzsinszky, J. P. Perdew, *Phys. Rev. Lett.* **2015**, *115*, 036402.
[26] (a) D. G. Truhlar, M. S. Gordon, *Science* **1990**, *249*, 491-498; (b) C. Gonzalez, H. B. Schlegel, *J. Phys. Chem.* **1990**, *94*, 5523-5527; (c) K. Fukui, *Acc. Chem. Res.* **1981**, *14*, 363-368; (d) K. Fukui, *J. Phys. Chem.* **1970**, *74*, 4161-4163.
[27] J. Xu, M. T. Rodgers, J. B. Griffin, P. B. Armentrout, *J. Chem. Phys.* **1998**, *108*, 9339-9350.
[28] J. A. M. Simoes, J. L. Beauchamp, *Chem. Rev.* **1990**, *90*, 629-688.
[29] L. M. Russon, S. A. Heidecke, M. K. Birke, J. Conceicao, M. D. Morse, P. B. Armentrout, *J. Chem. Phys.* **1994**, *100*, 4747-4755.
[30] D. E. Clemmer, J. L. Elkind, N. Aristov, P. B. Armentrout, *J. Chem. Phys.* **1991**, *95*, 3387–3393.
